# Extracellular Water and Phase Angle, Markers of Heightened Inflammatory State, and Their Extrapolative Potential for Body Composition Outcomes in Adults

**DOI:** 10.3390/metabo16010040

**Published:** 2026-01-02

**Authors:** Selma Cvijetić, Dario Boschiero, Hyehyung Shin, Andrew S. Reilly, Sarah T. Noorani, Nadja Vasiljevic, Jasminka Z. Ilich

**Affiliations:** 1 Division of Occupational and Environmental Health, Institute for Medical Research and Occupational Health, Ksaverska cesta 2, 10000 Zagreb, Croatia; cvijetic@imi.hr; 2 BioTekna, Marcon, 30020 Venice, Italy; dario.boschiero@biotekna.com; 3 IMC Gangnam Clinic, Seoul 06022, Republic of Korea; 4 College of Medicine, Florida State University Tallahassee, Tallahassee, FL 32306, USA; asr20e@med.fsu.edu (A.S.R.);; 5 Institute of Hygiene and Medical Ecology, Faculty of Medicine, University of Belgrade, Dr Subotica 8, 11000 Belgrade, Serbia; 6 Institute for Successful Longevity, Florida State University, 1107 West Call Street, Tallahassee, FL 32306, USA

**Keywords:** body composition, bioelectrical impedance analysis (BIA), extracellular-to-total body water ratio (ECW/TBW), phase angle (PhA), bone mass, muscle mass, fat mass

## Abstract

**Background/Aim:** Extracellular-to-total body water ratio (ECW/TBW) and phase angle (PhA, PA) reflect hydration and cellular health, but their relationship with bone, muscle, and fat, as primary components of body composition, is not fully elucidated. This study aimed to evaluate sex-specific differences in body composition and assess the diagnostic potential of ECW/TBW and PhA for identifying low bone/muscle mass, as well as increased fat mass, in generally healthy adults. **Methods**: This post hoc analysis utilized data from a multicenter, cross-sectional, Italian study (2010–2014) that included 20–90 years adults (n = 9717). Body composition was measured by bioelectrical impedance (BIA-ACC, BioTekna^®^), assessing bone, muscle mass, fat mass, ECW, TBW, and PhA. Low bone/muscle mass, as well as adiposity, were defined using standard cutoffs. Associations were examined using nonparametric tests and multiple regression analyses. **Results**: The mean age of men and women was similar (mean ~48 years). Men had significantly higher body mass index (BMI), intramuscular adipose tissue (IMAT%), T-score (bone), S-score (muscle), and PhA, while women had significantly higher fat mass (FM%) and ECW/TBW. ECW/TBW showed excellent discrimination for low muscle mass (AUC 0.845–0.922) and low bone mass (AUC 0.696–0.885), outperforming PhA. Neither ECW/TBW nor PhA reliably predicted increased fat mass. Regression models indicated ECW/TBW was strongly associated with age, sex, BMI, fat mass, and bone/muscle scores (R^2^ = 0.943), whereas PhA’s association was moderate (R^2^ = 0.368). **Conclusions**: ECW/TBW and PhA reflected sex-specific differences for body composition and effectively identified low muscle and bone mass (with better predictability of the former). Both showed a limited predictive ability for fat mass. Overall, both parameters provide complementary insights into sarcopenia and osteopenia and could be used for easy and non-invasive screening for these conditions.

## 1. Introduction

Body composition is a critical determinant of overall health, closely linked to metabolic, nutritional, and functional outcomes in both men and women [1,2,3,4]. Key components such as bone mineral density (BMD skeletal muscle mass, fat mass (FM, or adipose tissue) and adipose tissue distribution play central roles in identifying early pathophysiological changes, including osteoporosis, sarcopenia and obesity/adiposity —conditions often driven and/or initiated by chronic inflammation [5,6,7,8,9].

Inflammation is a tightly regulated and dynamic biological process involving the activation and coordination of immune cells, endothelial cells, and a network of molecular mediators that collectively function to eliminate injurious stimuli and initiate tissue repair [10]. In the context of acute inflammation, increased vascular permeability facilitates the extravasation of plasma proteins and leukocytes into the affected tissue, resulting in localized edema. Following resolution of the inflammatory trigger, interstitial fluid is typically cleared through lymphatic drainage and microvascular resorption mechanisms, thereby contributing to the restoration of tissue homeostasis [11]. When resolution fails or the injurious stimulus persists, chronic inflammation may develop. Chronic inflammation is characterized by sustained immune cell infiltration, ongoing release of pro-inflammatory cytokines, and often impaired lymphatic function [12], as well as by continuous capillary permeability, leading to extracellular water (ECW) accumulation [13]. ECW proportion to total body water (TBW) reflects fluid distribution between extracellular and intracellular compartments. The ECW/TBW elevation above commonly accepted thresholds (≥ 0.40) indicates inflammatory fluid shifts, tissue edema, and extracellular matrix expansion. Given these features, the ECW/TBW ratio have been proposed as surrogate markers of inflammation and fluid imbalance and is considered a physiological indicator for chronic inflammation [14]. Accordingly, individuals with chronic inflammatory conditions often show elevated ECW levels [15]. Additionally, elevated ECW/TBW ratio is commonly associated with reduced muscle mass, impaired physical function, and increased morbidity, particularly in older adults and those with chronic disease [16,17,18,19].

Phase Angle (PhA, PA) reflects cellular membrane integrity, body cell mass, intracellular water, and overall health status [20]. Higher PhA values indicate healthy, intact cell membranes and better nutritional status, while lower PhA values are frequently observed in individuals with inflammation, malnutrition, sarcopenia, or chronic disease [21,22]. Lower values (< 3.5°) are associated with impaired cellular function and are linked to chronic inflammatory states [22]. Oxidative stress and pro-inflammatory cytokines such as tumour necrosis factor alpha (TNF-α), interleukin-6 (IL-6), and C-reactive protein (CRP) impair membrane structure and function, consequently resulting in lower PhA [23,24,25], rendering the latter a good inflammatory marker.

Some newer studies have linked utility of PhA as a useful indicator for body composition components and their changes. For example, PhA was found to be a good indicator for muscle mass and physical performance in athletes, as well as in community dwelling older adults [26,27]. PhA was also applicable in reflecting weight changes during obesity treatments [28] and indicating the BMD status and the presence of osteoporosis in community dwelling adults [29,30]. In obesity or sarcopenic adiposity, chronic inflammation not only promotes fluid retention but also accelerates cellular and tissue degradation—evident through decreased PhA values and elevated ECW/TBW ratios [31,32].

Despite reflecting different physiological processes—fluid distribution (ECW/TBW) versus cellular integrity (PhA)—these two markers are interconnected. Both are shaped by inflammation and changes in body composition; elevations in ECW/TBW often coincide with reductions in PhA, pointing to shared disruptions in cellular and fluid homeostasis. Given their responsiveness to inflammation and oxidative stress, the ECW/TBW ratio has shown predictive value for clinical decline and compromised muscle function, particularly in aging populations, while PhA has been proposed as a prognostic biomarker for malnutrition [33], sarcopenia [34], and broader nutritional risk [35]. Therefore, metrics such as ECW/TBW and PhA are increasingly studied as indicators of hydration status, inflammation, nutritional adequacy, and cellular health [24,36], especially as both can easily be measured by bioelectrical impedance (BIA), a well-known non-invasive tool to assess body composition and related health risks.

Despite the growing use of ECW/TBW and PhA in clinical and research settings, their comparative diagnostic value for identifying body composition abnormalities and inflammation-related health risks has not been fully elucidated or studied. Most existing studies assess them independently, without directly comparing their associations with inflammation-driven changes in bone, muscle, and fat compartments. This is particularly true for the lack of comparative studies evaluating the diagnostic performance of these markers in predicting low bone or muscle mass or increased/redistributed adiposity in relation to inflammatory states. The aim of this study is to fill that gap by assessing sex differences in body composition and BIA-derived indices (ECW/TBW and PhA) in a generally healthy adult population (ages 20–90 years). Specifically, we evaluated the ability of ECW/TBW and PhA to predict low muscle and bone mass, as well as excess adipose tissue, and examine how physiological factors influence these associations through multivariate regression modelling. Understanding how ECW/TBW and PhA relate to basic body composition metrics and how these relationships differ by age, sex, or body composition compartments is crucial. Therefore, these markers hold potential to enhance early, sex-adapted screening and inform intervention strategies.

## 2. Materials and Methods

This is a retrospective analysis of the data originally collected as part of a large multicenter cross-sectional study in Italy between 2010 and 2014. The characteristics of the participants and the methodology have been described previously [37]. Below is a brief summary.

### 2.1. Participants

The study population included a convenience sample of Caucasian men and women aged 20 to 90 years who attended general medical practices across the country (total of 37). The primary objective was to evaluate bone health, body composition, and functional status in the general Italian population. All participants provided written informed consent and completed a comprehensive medical history questionnaire. The study received ethical approval from the Consortium for the Science and Technology Research AREA in Trieste, Italy, and was carried out in accordance with the ethical principles of the Declaration of Helsinki. Exclusion criteria included the presence of chronic illnesses (such as epilepsy, cancers, cardiovascular disorders, rheumatic conditions or psychiatric disorders), recent fractures (within the past 12 months), high alcohol consumption, use of bone-affecting treatments (e.g., steroids, bisphosphonates, calcium or vitamin D supplements, hormone therapies), pregnancy, and the presence of metal implants or electronic medical devices. The present post hoc analysis was approved by the Ethics Committee of the Institute for Medical Research and Occupational Medicine in Croatia (Approval No. 100-21/18-10).

### 2.2. Bioimpedance Measurements

Body composition was assessed using the dual frequency bioimpedance device BIA-ACC (BioTekna^®^, Marcon–Venice, Italy). Measurements were conducted with participants in a supine position, using four electrodes; two placed on the right wrist and two on the right ankle. The device measures bone mass and skeletal muscle mass yielding T-scores and S-scores, respectively, and fat mass (FM; as % of total body mass), intramuscular fat mass (IMAT; as percentage of FM), ECW, TBW and PhA (^0^). BIA–derived ECW/TBW and PhA were used as physiologic indicators of chronic, low-grade inflammation. ECW/TBW reflects fluid distribution between extracellular and intracellular compartments, and elevations above ≥ 0.40 indicate inflammatory fluid shifts, tissue edema, and extracellular matrix expansion. Phase angle, calculated from reactance and resistance between 50 kHz and 1 KHz, reflects cell membrane integrity, body cell mass, and intracellular water; lower values are associated with impaired cellular function and are linked to chronic inflammatory states. It is important to note that BIA-ACC provides a direct measurement of ECW at a low frequency of 1 kHz, and not as an estimate which could be obtained from classical bioimpedance devices. The ECW values measured by BIA-ACC have also previously been validated with classic inflammatory markers (e.g., cortisol, CRP) [38,39,40].

### 2.3. Diagnostic Criteria

The diagnostic criteria and cutoffs were based on the manufacturer’s reference values, as well as the current literature and clinical relevance. Accordingly, osteopenia/osteoporosis was diagnosed in participants with T-score < −1.0 for bone mass. Low muscle mass, reflecting sarcopenia, was identified based on the S-score cutoff of <−1.0. Overweight/obesity was diagnosed based on body fat of >25% for men and >30% for women. While the consensus for cutoff of >25% body fat for men is widely accepted, it is not the case for women. The recommended cutoffs range between 30% to 42% of body fat for overweight and obesity, respectively [41]. We opted for 32% based on the percentage that showed detrimental effect on bone in our previous study in postmenopausal women [7], and to align with subsequent recommendations [37,42]. The IMAT and PhA cutoffs were based on the manufacturer’s recommendation.

### 2.4. Statistical Analysis

The results are presented as mean ± standard deviation. The distribution of variables was tested using the Kolmogorov–Smirnov test. None of the variables were normally distributed; therefore, the difference in mean values of body composition parameters between men and women was tested using the Mann–Whitney test.

Receiver Operating Characteristic (ROC) curve analysis, based on logistic regression, was used to evaluate the diagnostic ability of two parameters, ECW/TBW and PhA, in detecting body composition deviations (from normal) in men and women. The Area Under the Curve (AUC) was calculated for each condition (low bone mass, low muscle mass, increased fat mass) to assess the discriminatory performance of each parameter. An AUC value close to 1.0 indicates excellent diagnostic accuracy, while an AUC of ≤0.5 suggests no better classification than random chance.

To compare the performance of two diagnostic models evaluated on the same subjects, we used the method proposed by Hanley and McNeil for comparing correlated ROC curves [43]. This approach applies a z-test based on the difference between the two AUCs, their standard errors, and an assumed correlation coefficient between them (*r* = 0.5). Two multiple regression models were created with ECW/TBW and PhA each, as dependent variables and body composition parameters as predictors. The resulting R^2^ explains the proportion of the variance in the dependent variable that is predictable from the independent variables. R^2^ between 0 and 1: indicates the percentage of variance explained (e.g., R^2^ = 0.943 means 94.3% of the variance is explained by the model). Multicollinearity was assessed using the Variance Inflation Factor (VIF). For both models (ECW/TBW and PhA as dependent variables), three predictors (BMI, FM and S-score) showed high collinearity (VIF > 10), but all were retained due to their established biological and clinical relevance. To assess the robustness of the regression results, cases with standard residuals exceeding ±3 were excluded and the analysis was rerun. In the model with ECW/TBW as dependent variable, the R^2^ decreased only slightly from 0.942 to 0.937, indicating that the model is largely robust and not excessively influenced by extreme cases. In the model with PhA as dependent variable, R^2^ increased from 0.368 to 0.756, reflecting the influence of extreme values.

An additional analysis was performed to examine the influence of age on body composition parameters. Participants were divided into age tertiles (≤42, 43–54, and ≥55 years), and differences in ECW/TBW and PhA between tertiles were assessed using one-way ANOVA followed by Tukey’s post hoc test. All analyses were performed using Statistica, version 15 (TIBCO Software Inc. Palo Alto, CA, USA). The level of significance in all analyses was set at *p* < 0.05.

## 3. Results

Women and men were of a similar mean age (47.6 vs. 47.8 years, respectively) (Table 1). The BMI in women was close to the upper reference (normal), while in men it was slightly elevated. Men had significantly higher values for weight, height, BMI, and IMAT% compared to women, with all differences being statistically significant (*p* < 0.001). Fat mass percentage (FM%) was slightly higher in women compared to men, with the statistically significant difference (*p* < 0.001). However, a higher proportion of men exceeded the reference values for FM% and IMAT% than women. The mean T-score and S-score values were within the normal reference range for each sex, with the exception of a borderline reduced T-score (−1.1) in women. However, both bone and skeletal muscle parameters showed notable and expected sex-based differences: men had significantly higher T-score and S-score values than women (*p* < 0.001 for both). ECW/TBW ratio, was significantly higher in women compared to men (*p* < 0.001). Men showed significantly higher phase angle values (*p* < 0.001). However, mean PhA was lower and mean ECW/TBW was higher compared to normal reference, in both sexes.

**Figure 1:**
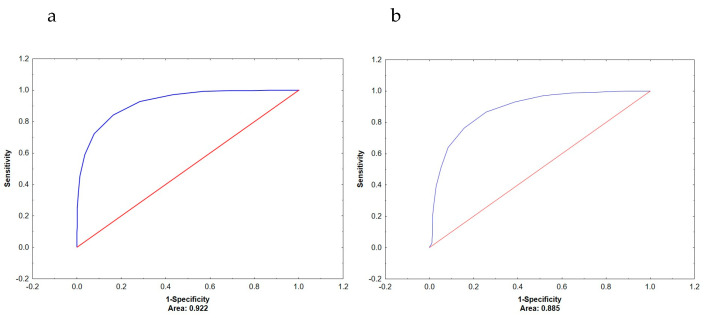
ROC curve for ECW/TBW-based prediction of low muscle mass (**a**) and low bone mass (**b**) in women. ROC = receiver operator characteristics; CW/TBW = extracellular water total body water ratio; AUC = area under the curve.

The diagnostic performance of ECW/TBW and PhA in predicting low muscle mass, low bone mass, and increased fat mass was assessed separately for women and men using the area under the ROC curve (AUC). The results are summarized in Table 2. Among women, ECW/TBW demonstrated great discriminatory ability for identifying low muscle mass (AUC = 0.922) and low bone mass (AUC = 0.885), significantly outperforming PhA in both cases (*p* < 0.001). Figure 1a,b. Neither ECW/TBW nor PhA proved to be good predictors of increased FM%, although ECW/TBW showed a moderately better AUC (0.713) compared to PhA (0.574), with the difference being statistically significant (*p* < 0.001).

**Figure 2:**
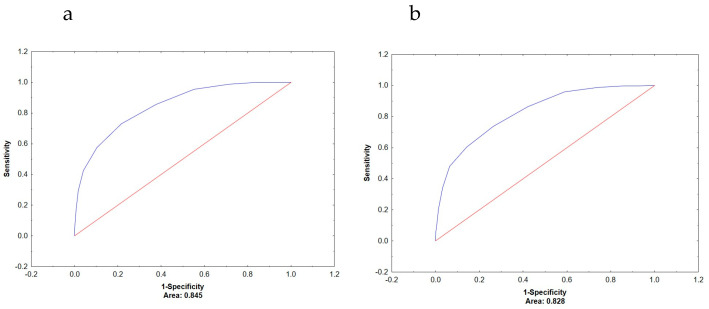
ROC curve for ECW/TBW-based prediction of low muscle mass (**a**) and low bone mass (**b**) in men. ROC = receiver operator characteristics; ECW/TBW = extracellular water total body water ratio; AUC = area under the curve.

In men, ECW/TBW also showed superior AUC values compared to PhA in detecting low muscle mass (0.845 vs. 0.719) and low bone mass (0.828 vs. 0.696), again with statistically significant differences (*p* < 0.001) Figure 2a,b. However, when predicting increased fat mass, the AUC values for ECW/TBW (0.647) and PhA (0.650) were nearly identical, and the difference was not statistically significant (*p* = 0.920).

Multiple linear regression models were conducted to identify association of ECW/TBW ratio and PhA with body composition parameters (Table 3). The model predicting ECW/TBW showed a high explanatory power (R^2^ = 0.943), indicating that 94.3% of the variance in ECW/TBW was explained by the included variables, with coefficients remaining stable after exclusion of extreme cases (R^2^ decreased only slightly from 0.943 to 0.937). All associations in the model were statistically significant (*p* < 0.001). When controlling for age and sex, ECW/TBW was positively associated with BMI, and negatively associated with sex, fat mass, S-score, and T-score. These findings suggest that higher age and higher BMI are associated with increased ECW/TBW, while male sex, greater bone density, and higher fat mass are associated with lower ECW/TBW values. Moreover, our findings show a clear age-related trend, with ECW/TBW increasing with age (45.4, 47.4, 49.9 for youngest, middle, and oldest tertiles, respectively), and PhA decreasing across age tertiles (2.9, 2.6, 2.0), (*p* < 0.001; ANOVA). The regression model for PhA explained a smaller proportion of the variance (R^2^ = 0.368). However, robustness analysis increased R^2^ from 0.368 to 0.756, reflecting the influence of extreme values. When controlling for age and sex, PhA was positively associated with FM%, T-score and S-score, while it was negatively associated with BMI (*p* < 0.001 for all predictors). These results indicate that although both models identified age, sex, BMI, fat mass, muscle and bone scores as significant predictors, the ECW/TBW was stronger determined by these factors than PhA, as reflected in the substantially higher R^2^ value for the former.

## 4. Discussion

This study revealed notable sex-related differences in body composition and bioimpedance parameters among a large sample of generally healthy adults, of which some were expected. Despite similar mean ages, men exhibited significantly higher values for body weight, height, BMI, and IMAT%, while women showed higher FM% values, consistent with known physiological differences in fat distribution and muscle mass between sexes [44,45]. Although average T-score and S-score values were within the normal range in both sexes, women had a borderline T-score, which is in line with the higher prevalence of osteopenia and osteoporosis generally observed in female populations [46,47]. Similarly, a recent study in young adults revealed the importance of taking into account both sex- and obesity-specific factors when evaluating body composition and developing strategies to maintain or improve some of the body composition components, particularly the bone health [48]. In that study, non-obese individuals with higher levels of FM showed better bone tissue quality, assessed by the speed of sound (SOS). Furthermore, age and visceral FM were significant predictors of SOS in both non-obese men and obese women, but with opposite effects: visceral FM positively predicted SOS in non-obese men, whereas it negatively predicted SOS in obese women.

The ECW/TBW and PhA also demonstrated distinct sex-specific trends–both findings consistent with literature suggesting that women, compared to men, typically have a higher extracellular water proportion and lower cellular integrity as reflected by PhA [18,49]. Importantly, both ECW/TBW and PhA were already altered in both sexes, potentially indicating early changes in tissue composition and hydration status, associated with aging and metabolic adjustments [25,50]. ROC analysis revealed the diagnostic utility of ECW/TBW, particularly in detecting low muscle and bone mass in both sexes, outperforming PhA in these domains. In women, ECW/TBW showed excellent discrimination for both low muscle mass (AUC = 0.922) and low bone mass (AUC = 0.885), and also outperforming PhA in men. There is a lack of studies directly comparing the associations of ECW/TBW and PhA with bone mass, muscle mass, and adipose tissue. One study reported that PhA reflects lean mass and cellular integrity, whereas ECW/TBW ratio captures fluid imbalance and adiposity [51]. Japanese researchers also demonstrated that ECW/TBW ratios were significantly higher in elderly men with reduced muscle function, indicating its utility in assessing sarcopenia [52]. Besides reporting that ECW/TBW increases with age these researchers also found that it is typically higher in women than in men, suggesting both age- and sex-related changes in fluid distribution and tissue composition, similarly as the results of our study showed. Another study revealed that individuals with ECW/TBW ≥ 40% had significantly higher prevalence of sarcopenia compared to those with lower ratios, suggesting a potential role of extracellular fluid imbalance in musculoskeletal decline [50]. All these findings, together with ours, highlight the relevance of fluid distribution metrics in the assessment of age-related body composition changes.

It is well known that chronic inflammation disrupts the bone remodeling, increasing bone resorption and consequently heightens the risk of osteoporosis [7,29,30,53]. Among other things, inflammation also alters the ECW distribution, and a higher ECW/TBW ratio may thus serve as a surrogate marker for such inflammatory states or hydration imbalances, both of which can adversely affect bone and muscle health [54]. Adipose tissue, particularly visceral fat, plays a central role in this inflammatory environment by secreting adipokines and cytokines that exacerbate systemic inflammation. In turn, this feedback loop contributes to further fluid retention and ECW expansion [55]. Additionally, chronic inflammation and adiposity may impair capillary permeability and lymphatic drainage, leading to a redistribution of fluids from intracellular to extracellular compartments [56]. According to these findings, it is suggested that ECW/TBW, due to its sensitivity to shifts in body fluid compartments and inflammation, could potentially outperform PhA as a marker for musculoskeletal deterioration, especially in aging populations [1], as found in our study.

Regarding the adipose tissue, both ECW/TBW and PhA showed limited ability to predict excess fat mass in our study, as reflected by modest ROC-AUC values, indicating low to moderate discriminatory performance. Although our results showed a positive association between fat mass and PhA after adjusting for confounding factors, this finding likely reflects population-specific characteristics, such as metabolic health and fat distribution (e.g., visceral vs. subcutaneous fat), rather than a direct relationship between PhA and adiposity per se. Barbosa-Silva et al. reported that higher PhA values may be associated with greater subcutaneous fat and preserved cell membrane integrity, particularly in individuals without chronic illness [18]. Conversely, other studies in populations with obesity or sarcopenia reported an inverse relationship between PhA and fat mass, highlighting the complexity and context-dependence of this marker [28]. However, both indicators may hold clinical relevance when used as part of a comprehensive body composition assessment, particularly given their utility in evaluating hydration status, cellular function, and the broader metabolic context of fat accumulation, rather than as standalone markers of adiposity. Conversely, the association between ECW/TBW and fat mass likely reflects alterations in fluid distribution relative to fat-free mass, rather than changes in adipose tissue itself.

The results from multiple regression models in our study supported the findings that ECW/TBW is influenced by key body composition parameters stronger than PhA, as reflected in higher explanatory power of the former. Katsura and colleagues found that the association between PhA and mortality in patients with cachexia was largely mediated by the ECW/TBW ratio and that adjusting PhA for the ECW/TBW ratio may improve its predictive value [57]. Overall, ECW/TBW appears to be a more sensitive and informative marker than PhA for identifying unfavorable changes in muscle and bone mass, but less so in fat mass. These findings support the growing evidence advocating for its inclusion in body composition assessments alongside or even in place of PhA [17,50,58]. Our age-stratified analyses revealed clear differences in body composition parameters across age tertiles. This is consistent with the regression results, highlighting that age is an important determinant of fluid distribution and cellular integrity. However, although age and sex were statistically significant predictors of ECW/TBW, their regression coefficients were relatively small, indicating limited practical impact compared with body composition variables, which accounted for most of the explained variance.

There are several limitations, as well as strengths, of this study: the cross-sectional design limits causal interpretation, and the findings may not be generalizable to other clinical populations with some chronic diseases or to other ethnicities. Additionally, the utility of ECW/TBW and PhA may vary depending on the individual’s hydration status, menstrual cycle in women, and time of the day when measurements were taken (information lacking in the dataset). We also did not have the information for the dietary intake or physical activity of the participants, neither the measurements of some other inflammatory markers like CRP, IL-6, TNF-alpha. Although BIA-ACC technology has been validated for bone and inflammatory markers, it does have its limitations, but because of its portability and ease of use, it is very convenient for large population measurement. In the TIBCO Statistica versions 15 (software available for this analysis), the Generalized Linear Models part does not provide automatic cut-off values or Youden index tables. As a result, it was not possible to extract fixed thresholds directly from logistic regression results. This limitation is inherent to the software, reflecting a technical restriction rather than a methodological oversight. Despite the limitations, key unique aspect of this study is its comprehensive approach, encompassing all three major body composition compartments: bone, muscle, and fat which allowed for a more nuanced interpretation of how hydration status and cellular health and integrity, reflected by ECW/TBW and PhA, predicted osteopenia and sarcopenia. Additionally, since the study included large sample size of almost 10,000 generally healthy individuals of homogenous ancestry, the statistical power, reliability, and generalizability of the findings were strengthened.

## 5. Conclusions

This study provides the insights into sex-specific differences in body composition (bone, muscle, adipose tissue) and bioimpedance markers among healthy adults, highlighting the diagnostic potential of ECW/TBW and PhA. Both ECW/TBW and PhA are valuable BIA-derived parameters (especially the former) in identifying low muscle and bone mass and to a lesser degree fat mass (only in women). The borderline T-scores found in women indicate that bone health in this group may already have been compromised. Therefore, incorporating both measures can provide a more comprehensive, non-invasive risk assessment and screening for sarcopenia, osteopenia and, to a certain degree adiposity, in this population segment. Additionally, our results reveal the clinical potential for the assessment of bone and muscle deficits, and to some extent body fat excess, that could be easily obtained in general population utilizing a noninvasive and rapid technology as offered by BIA.

## Figures and Tables

**Table 1:** Age, anthropometry and bioimpedance-derived parameters in participants.

Variables	Mean ± SD		*p* *	Range	Percentile 25–75%	Reference Values
	Women (6412)	Men (3305)				
Age (yrs)	47.6 ± 13.5	47.8 ± 14.1	0.430	20.0–90.0	38.0–57.0	/
Weight (kg)	66.7 ± 14.4	82.9 ± 15.1	<0.001	40.0–149.0	60.0–82.0	/
Height (cm)	163.1 + 7.0	176.4 ± 7.0	<0.001	141.0–199.0	160.0–174.0	/
BMI (kg/m^2^)	25.1 ± 5.4	26.6 ± 4.6	<0.001	14.5–57.2	21.9–28.3	18.5–29.9
T-score	−1.1 ± 0.8	−0.3 ± 0.7	<0.001	−3.3–5.2	−1.5-(−0.3)	>−1
S-score	−0.9 ± 1.4	−0.1 ± 1.2	<0.001	−4.5–9.0	−1.6–0.1	>−1
FM (%)	33.2 ± 8.6	32.5 ± 7.3	<0.001	5.0–57.0	27.0–39.0	<30.0 (F) <25.0 (M)
IMAT (%)	2.0 ± 0.5	2.2 ± 0.4	<0.001	0–3.5	1.7–2.5	<2%
ECW/TBW (%)	50.0 ± 4.3	42.7 ± 2.7	<0.001	36.0–71.0	43.0–51.0	40%
PhA (^0^)	2.3 ± 1.3	3.0 ± 1.3	<0.001	−2.1–22.7	1.8–3.4	>3.5

* Mann–Whitney test; BMI = body mass index; FM = fat mass; IMAT = intramuscular adipose tissue; ECW = extracellular water; TBW = total body water; PhA = phase angle.

**Table 2:** Differences between the two AUC in women and men.

		Area Under the Curve (AUC)		
		ECW/TBW	PhA	*p* *
Women	Low/normal muscle mass	0.922	0.608	<0.001
	Low/normal bone mass	0.885	0.577	<0.001
	Increased/normal fat mass	0.713	0.574	<0.001
Men	Low/normal muscle mass	0.845	0.719	<0.001
	Low/normal bone mass	0.828	0.696	<0.001
	Increased/normal fat mass	0.647	0.650	0.920

* Hanley and McNeil method; ECW = extracellular water; TBW = total body water; PhA = Phase angle.

**Table 3:** Association of ECW/TBW and PhA with fat mass, skeletal muscle mass and bone mass parameters.

	Dependent variables			
	ECW/TBW		PhA	
	b	*p* *	b	*p* *
Intercept	47.8	<0.001	10.1	<0.001
Age	0.14	<0.001	0.01	<0.001
Sex	−6.18	<0.001	0.82	<0.001
BMI	0.46	<0.001	−0.45	<0.001
T-score	−1.14	<0.001	1.22	<0.001
S-score	−1.58	<0.001	2.13	<0.001
FM%	0.38	<0.001	0.08	<0.001
R^2^	0.943		0.368	

* Multiple regression (b = coefficient of regression). ECW = extracellular water; TBW = total body water; PhA = Phase angle; BMI = body mass index; FM = fat mass; R^2^ = coefficient of determination.

## Data Availability

The raw data supporting the conclusions of this article will be made available by the authors upon request, with the signed copyrights form.

## Abbreviations

The following abbreviations are used in this manuscript:

ACC	Alternating Current Conductivity
AUC	Area Under the Curve
BIA	Bioelectrical Impedance Analysis
BIA-ACC	Bioelectric Impedance Analyzer-Alternating Current Conductivity
BMD	Bone Mineral Density
BMI	Body Mass Index
CRP	C-reactive Protein
ECW	Extracellular Water
ECW/TBW	Extracellular Water-to-Total Body Water Ratio
FM	Fat Mass
FM%	Fat Mass Percentage
IL-6	Interleukin-6
IMAT	Intramuscular Adipose Tissue
IMAT%	Intramuscular Adipose Tissue Percentage
PhA or PA	Phase Angle
ROC	Receiver Operator Characteristic
S-Score	Skeletal Muscle Score
TBW	Total Body Water
TNF-α	Tumor Necrosis Factor Alpha
T-Score	Bone Density Score
